# Correlation Between Ultrasonographic Inferior Vena Cava Indices and Post-subarachnoid Anesthesia Hypotension: A Prospective Study

**DOI:** 10.7759/cureus.83215

**Published:** 2025-04-29

**Authors:** Mario A Leotau Rodriguez, Andrea Juliana Castillo Nino, Angy Valenzuela

**Affiliations:** 1 Anesthesiology and Perioperative Medicine, Clínica del Dolor Aliviar, Fundación Oftalmológica de Santander – Clínica Carlos Ardila Lulle (FOSCAL) Universidad Autónoma de Bucaramanga (UNAB), Floridablanca, COL; 2 Anesthesiology and Perioperative Medicine, Fundación Oftalmológica de Santander – Clínica Carlos Ardila Lulle (FOSCAL) Universidad Autónoma de Bucaramanga (UNAB), Floridablanca, COL; 3 General Physician, Universidad Industrial de Santander, Bucaramanga, COL

**Keywords:** arterial hypotension, hemodynamic monitoring, inferior vena cava, subarachnoid block, ultrasonography

## Abstract

Introduction: Arterial hypotension is the most common adverse event following subarachnoid block (SAB). Numerous studies have attempted to predict its occurrence, but the results remain inconclusive. The use of 'preloading' with intravenous fluids as a preventive measure remains controversial. Ultrasonographic assessment of the inferior vena cava (IVC) may be useful for evaluating volemia; however, its role as a predictor of hypotension in SAB has not been thoroughly investigated.

Objective: To describe the correlation between IVC parameters derived (IVC-PD), the collapsibility index (IVCCI), and the distensibility index (IVCDI), and the development of hypotension following SAB.

Methods: This was a prospective, analytical study of diagnostic technology involving 70 patients undergoing SAB. Baseline ultrasonographic measurements of IVC parameters were recorded. The evaluated IVC parameters included the end-expiratory diameter (IVCe > 2 cm), IVCCI (≥ 40%), and IVDI (≥ 18%). Hypotension was defined as systolic blood pressure (SBP) < 90 mmHg, mean arterial pressure (MAP) < 60 mmHg, or a decrease in SBP or MAP ≥ 25% in patients with arterial hypertension. Correlation strength was evaluated using Spearman's index (rho), and simple and binomial linear regression analyses were performed to assess other factors associated with hypotension.

Results: The correlation between IVC parameters and post-SAB hypotension was weak and negative (rho = -0.058). Linear regression analysis did not yield statistically significant results (*p* > 0.05), and the coefficients obtained were negative. A history of arterial hypertension and a sensory block level below T4 were identified as risk factors for hypotension.

Discussion: The incidence of arterial hypotension was 27.14%, aligning with the upper range of previous studies. Risk factors such as a sensory block above T4 and a history of arterial hypertension were significantly associated with hypotension. However, no significant correlation was found between pre-anesthetic IVC-PD measurements and post-SAB hypotension, consistent with some prior findings. Variability across studies may relate to differences in patient characteristics and ventilation status.

Conclusion: While confirming established hypotension risk factors, this study did not support IVC-based prediction, contrasting with some literature. Further research should address limitations like sample size and single-center design to clarify IVC’s role in spontaneously ventilating patients.

## Introduction

The incidence of arterial hypotension during subarachnoid block (SAB) ranges from 30% to 90%, with such wide variability stemming from multiple studies conducted on obstetric and nonobstetric populations. Numerous risk factors have been associated with this condition, and various management strategies have been proposed with highly variable outcomes [1-3]. Hypotension has been linked to sympathetic blockade induced by SAB, and intravenous fluid therapy has almost always been the first-line intervention [4].

Currently, noninvasive blood pressure (BP) monitoring is routinely used during SAB [1]. However, it has limitations, including delays in obtaining real-time data and managing episodes of arterial hypotension [5]. Furthermore, many protocols rely on “volume loading” to prevent or manage hypotension, often based on an assumption of unconfirmed hypovolemia. For this reason, in recent years, diagnostic approaches using noninvasive imaging methods that provide real-time information have been studied [6,7].

Ultrasound (US) is a feasible imaging method for initial management. It offers a diagnostic tool to evaluate hemodynamic variations, responsiveness to fluid therapy, and intravenous fluid management [8]. The utility of inferior vena cava (IVC) measurements lies in their ability to correlate with the patient’s volemic state. This is based on the vessel’s collapsibility and its direct relationship with right ventricular heart function. It is well established that the diameter and the IVC collapsibility index (IVCCI) are directly associated with intravascular volume, with greater accuracy than other methods such as blood pressure and pulse pressure [9].

However, the correlation between IVC measurements and hemodynamic variations has not been established. This includes blood pressure changes in alert patients undergoing SAB. The objective of this study is to describe the correlation between IVC parameters derived (IVC-PD), IVCCI, and IVC distensibility index (IVCDI), and the development of hypotension following SAB.

## Materials and methods

This was a prospective, analytical study evaluating diagnostic technology. It was conducted at Fundación Oftalmológica de Santander - Clínica Carlos Ardila Lulle (FOSCAL), located in Floridablanca, Colombia. The study included ambulatory and hospitalized patients over 18 years of age who were scheduled for surgery, underwent SAB with American Society of Anesthesiologists (ASA) I or II, and agreed to participate by signing informed consent. Exclusion criteria included pregnant patients, those with poor acoustic windows (inadequate visualization of the IVC or inability to identify its anterior and posterior borders), and patients with ASA III or higher.

A nonprobabilistic consecutive sampling method was used, including all patients who met the inclusion criteria. Data collection took place from July 1, 2020, to January 2021, resulting in a sample of 70 patients.

This study was approved by the institution's ethics committee under approval number 03829.

Study variables

The dependent variable analyzed was the degree of correlation between IVC variability indices and the development of arterial hypotension. Hypotension was defined as either a systolic blood pressure (SBP) < 90 mmHg, a mean arterial pressure (MAP) < 60 mmHg, or a >25% decrease in SBP from baseline in patients with arterial hypertension [10]. The degree of correlation was assessed using Spearman’s index (rho). Interpretation of the Spearman rho coefficient follows these principles: values close to one indicate a strong positive correlation, values close to -1 indicate a strong negative correlation, and values near 0 indicate no linear correlation.

Monitoring parameters were assessed by the principal investigator, who received prior training for this purpose. Measurements were performed using a Sonosite Edge/Turbo US device (FUJIFILM Sonosite, Inc., Bothell, WA), considering the cutoff points proposed by the American Society of Echocardiography (Table 1). These cutoff points include 18% variability in the diameter of the IVC as an indicator for distinguishing between volume-responsive and non-responsive patients, with a sensitivity and specificity of 90% [11]. 

**Table 1:** Cutoff points and interpretation of IVC-PD IVCCI: inferior vena cava collapsibility index; IVCDI: inferior vena cava distensibility index; IVC: inferior vena cava; IVC-PD: IVC parameters derived

Parameter	Cutoff point	Interpretation
IVCCI	> 40%	Responds to volume
	< 40%	Does not respond to volume
IVCDI	> 18%	Responds to volume
	< 18%	Does not respond to volume
IVC diameter at end-inspiration	Diameter < 2 cm and inspiratory collapse > 50%	Hypovolemia
	Diameter ≥ 2 cm and inspiratory collapse < 50%	No hypovolemia

The independent variables collected included age, sex, weight, height, surgical procedure, ASA classification, condition as defined by ASA, New York Heart Association (NYHA) classification, use of intrathecal opioids, subarachnoid opioid dose, subarachnoid hyperbaric bupivacaine dose, use of antihypertensive medications, consumption of antihypertensive medications, use of vasopressor medications, vasopressor medication dose, use of crystalloids, level of lumbar puncture, patient position, and sensory block level.

Data collection

Sociodemographic and clinical variables were recorded using a data collection instrument based on the patient’s electronic medical record and the monitoring data established by their treating physician. Measurements of the IVC diameters during inspiration and expiration were taken from the subxiphoid window. The patient was positioned in the supine position, and a low-frequency sector or convex transducer was used. The IVC was located using two-dimensional US in its right paramedian position, anterior to the spine, distinguishable from the aorta by its thinner wall.

The IVC diameter was measured in M-mode, 2 cm from the IVC-right atrium junction. The maximum diameters during inspiration and expiration were recorded, with a correction factor applied to ensure measurements were taken along the transverse axis of the IVC. 

These measurements were taken before administering SAB. BP measurements were performed automatically using a noninvasive BP device upon the patient’s admission to the operating room. Follow-up monitoring was conducted after the anesthetic procedure.

Data management and statistical analysis

The data recorded were tabulated in an Excel 2013 (Microsoft® Corp., Redmond, WA) database and exported to STATA 14.0 (StataCorp LLC, College Station, TX) for analysis. Patients were classified into two groups based on the presence or absence of arterial hypotension. The data were described using measures of frequency, central tendency, and dispersion. To assess the correlation of numerical variables, the Shapiro-Wilk test was applied to confirm normal distribution. Consequently, the nonparametric Spearman’s rank correlation coefficient test was used. A p-value < 0.05 was considered significant, with a 95% confidence level and an alpha error of 5%.

A simple linear regression model was performed to evaluate the dependent relationship between IVC-PD and arterial hypotension. The correlation coefficient was interpreted according to established parameters. The simple linear regression model was applied to explore the dependency relationship between IVC-PD and arterial hypotension.

## Results

A total of 70 patients were evaluated. The mean age was 58.31 years (range: 19-89 years). Among comorbidities, arterial hypertension was present in 34.29% (n=24) of patients. The remaining demographic and clinical characteristics are shown in Table 2.

**Table 2:** Sociodemographic variables kg: kilograms; m: meters; BMI: body mass index

Variable	Average (SD); Min – Max
Age (years)	58.31 (18.2); 19 – 89
Weight (kg)	71.74 (12.15); 45– 98
Height (m)	1.66 (0.09); 1.40 – 1.85
BMI	25.78 (3.69); 16 – 37

The most frequent type of surgery was general abdominal surgery, accounting for 48.57% (n=34), followed by orthopedic hip and knee joint replacement surgery at 34.29% (n=24) and lower extremity vascular surgery interventions at 17.14% (n=12). Regarding the anesthetic technique, the puncture level was performed at the intervertebral space L3-4 in 81.43% (n=57) of cases, followed by L4-5 in 15.71% (n=11) and L2-3 in 2.86% (n=2). The intrathecal anesthetic used in all cases was 0.5% hyperbaric bupivacaine, with a variable dose ranging from 12.5 to 17 mg in 78.57% (n=55) of cases and 8-10 mg in 21.43% (n=15) of patients. The use of opioids was recorded in 18.57% (n=13) of cases. Preload administration was performed in only 8.57% (n=6) of cases.

The seated position was the most commonly used for SAB administration, at 92.86% (n=65), achieving a variable sensory block level from T5-T10 in 85.71% (n=60) of cases and T4 in 14.29% (n=10). The overall incidence of arterial hypotension was 27.14%, with a 95% confidence interval of 16.42 - 37.82. The highest incidence of the event occurred during minutes five and eight, with rates of 11.43% (n=8) and 7.14% (n=5), respectively (Table 3).

**Table 3:** Incidence of arterial hypotension CI: confidence interval

Time point	Hypotension % (n)	95% CI
Minute 1	0%	-
Minute 3	5.71% (4)	0.13 – 11.28
Minute 5	11.43% (8)	3.78 – 19.06
Minute 8	7.14% (5)	1.04 – 21.81
Minute 10	4.29% (3)	0.57 – 9.14
Minute 15	4.29% (3)	0.57 – 9.14
Minute 20	2.86% (2)	1.14 – 6.85

When evaluating arterial hypotension according to operational variables, there was variability in the incidence (14.29% to 17.14%, p > 0.05). When using a variable involving the use of vasopressors as a surrogate for hypotension, the incidence of arterial hypotension increased from 17.14% (n=12) to 25.71% (n = 18). If the use of vasopressors was detected and the patient did not record values consistent with hypotension, it was assigned as an event (Table 4).

**Table 4:** Incidence of hypotension according to operational definitions SBP: systolic blood pressure; MAP: mean arterial pressure; CI: confidence interval

Parameters	Hypotension % (n)	95% CI
SBP < 90 mmHg	15.71% (11)	6.9 – 24.4
MAP < 60 mmHg	14.29% (10)	5.88 – 22.68
SBP < 90 and/or MAP < 60	17.14% (12)	8.1 – 26.19
Hypertensive patients with SBP < 25% of baseline	17.14% (12)	8.1 – 26.19
Use of vasopressors	25.71% (18)	15.21 – 36.21
General hypotension incidence	27.14% (19)	16.42 – 37.82

In the binomial regression analysis conducted to evaluate potential factors associated with the development of post-SAB hypotension, significant variables were documented, such as the history of arterial hypertension (odds ratio (OR) 2.12, p = 0.0484, 95% CI = 1.002 - 4.52) and the level reached at T4, with an elevated OR (OR 3.5, p = 0.004, 95% CI = 1.82 - 6.69).

The general average diameter of the IVC in inspiration and expiration was less than 2 cm, and the collapse of the IVC was greater than 50% (60.42%), categorizing the variable as "hypovolemia." The average value of the IVCDI was above the cutoff point (50.27% vs. 18%), unlike the IVCCI, which was near the cutoff point (39.54% vs. 40%) (Table 5).

**Table 5:** IVC parameters derived (IVC-PD) IVC: inferior vena cava; IVCCI: inferior vena cava collapsibility index; IVCDI: inferior vena cava distensibility index; SD: standard deviation

IVC-PD	Average	Cutoff points	SD	Min – Max
IVC diameter Inspiration	1.00	< 2 cm	0.35	0.4 – 2.02
IVC diameter expiration	1.65	< 2 cm	0.39	0.8 – 2.63
IVC collapse	60.42	> 50%	13.43	29 – 87
IVCCI	39.54	> 40%	13.39	13.16 – 70.91
IVCDI	50.27	> 18%	19.39	14.08 – 94.93

The US evaluation of the IVC allowed for the classification of patients into responders (hypovolemic) and nonresponders, according to previously defined and validated cutoff points (Table 6).

**Table 6:** Arterial hypotension and IVC parameters derived (IVC-PD) IVC: inferior vena cava; IVCCI: inferior vena cava collapsibility index; IVCDI: inferior vena cava distensibility index

Parameters	Cutoff point	Responders % (Fr)	Hypotension in responders	p-value
IVC diameter Inspiration + IVC collapse	< 2 cm and > 50%	77.14% (54)	25.92% (14)	0.674
IVCCI	≥ =40	50% (35)	25.71% (9)	0.788
IVCDI	≥ =18	62.85% (44)	26.86% (18)	0.805

The correlation between hypotension and all IVC-PD was very weak and negative for the indices of distensibility and collapsibility (rho 0.05 and 0.06). Similarly, it was weakly positive for the other IVC-PD (Table 7).

**Table 7:** Correlation indices between IVC parameters derived (IVC-PD) and hypotension IVC: inferior vena cava; IVCCI: inferior vena cava collapsibility index; IVCDI: inferior vena cava distensibility index

	Hypotension	IVC diameter Inspiration	IVC diameter Expiration	IVC Collapse	IVCCI	IVCCI
Hypotension	1					
IVC diameter Inspiration	0.1037	1				
IVC diameter expiration	0.0748	0.7371	1			
IVC collapse	0.0536	0.7476	0.1452	1		
IVCCI	-0.0582	-0.7491	-0.1461	-0.9994	1	
IVCDI	-0.0582	-0.7491	-0.1461	-0.9994	1	1

No statistical significance was obtained in the regression analysis. Similarly, all the coefficients obtained were negative, except for the IVCCI, which was weakly positive (Table 8).

**Table 8:** Regression analysis for hypotension and IVC-PD IVC: inferior vena cava; IVCCI: inferior vena cava collapsibility index; IVCDI: inferior vena cava distensibility index; CI: confidence interval

Hypotension	Coefficient	Standard Error	p-value	95% CI
IVC diameter inspiration	34.11	55.14	0.538	(-76.1 – 144)
IVC diameter expiration	-14.96	34.63	0.667	(-84.1 – 54)
IVC collapse	4.42	9.28	0.635	(-14.1 – 22)
IVCCI	4.04	9.60	0.675	(15.1 – 23)
IVCDI	0.55	1.11	0.616	(-1.6 – 2)
-Constant	-354.4	932.9	0.705	(-2218.1 – 1509)

**Figure 1:**
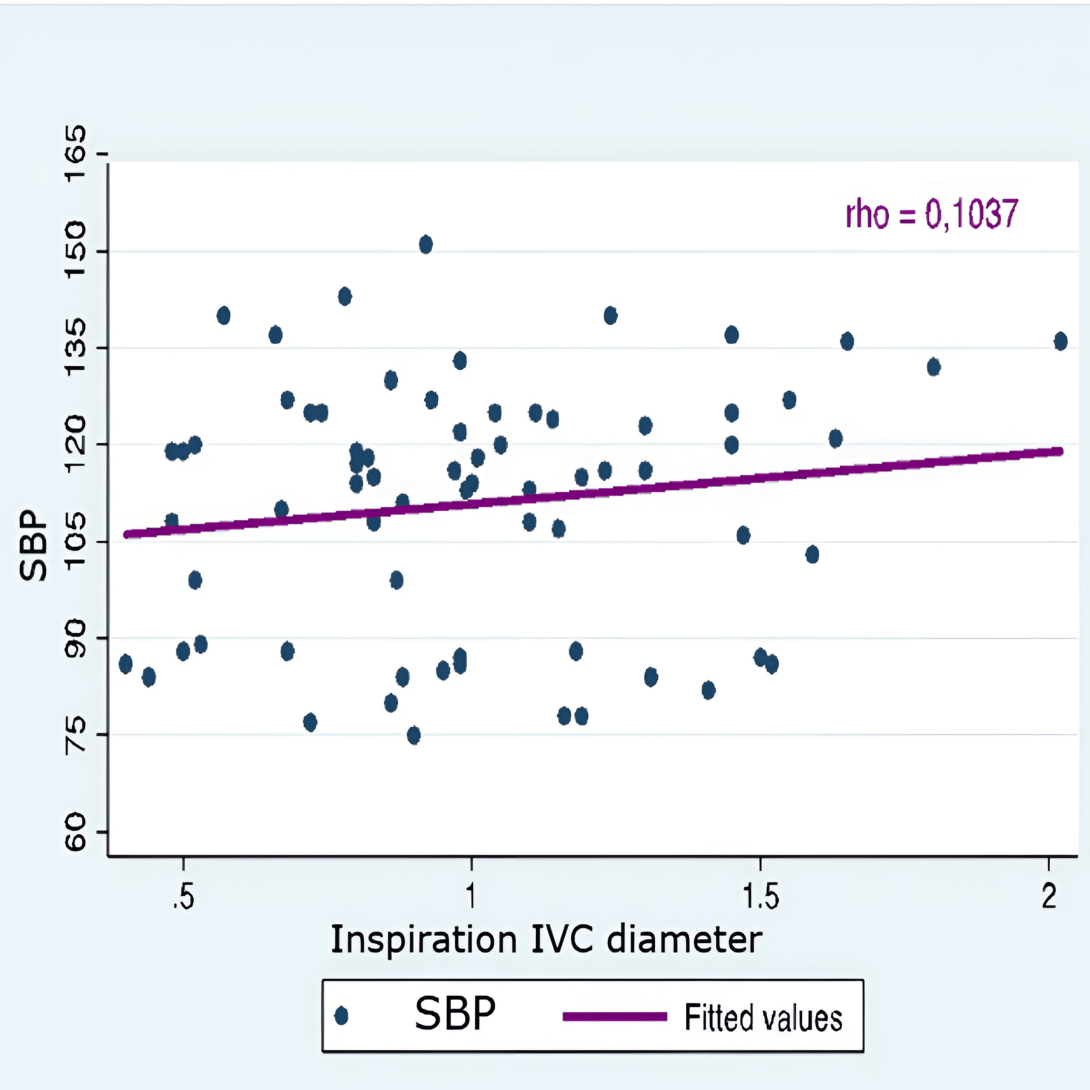
Correlation SBP and inspiration IVC diameter SBP: systolic blood pressure; IVC: inferior vena cava; Y axis: SBP (mmHg); X axis: inspiration IVC diameter (cm)

**Figure 2:**
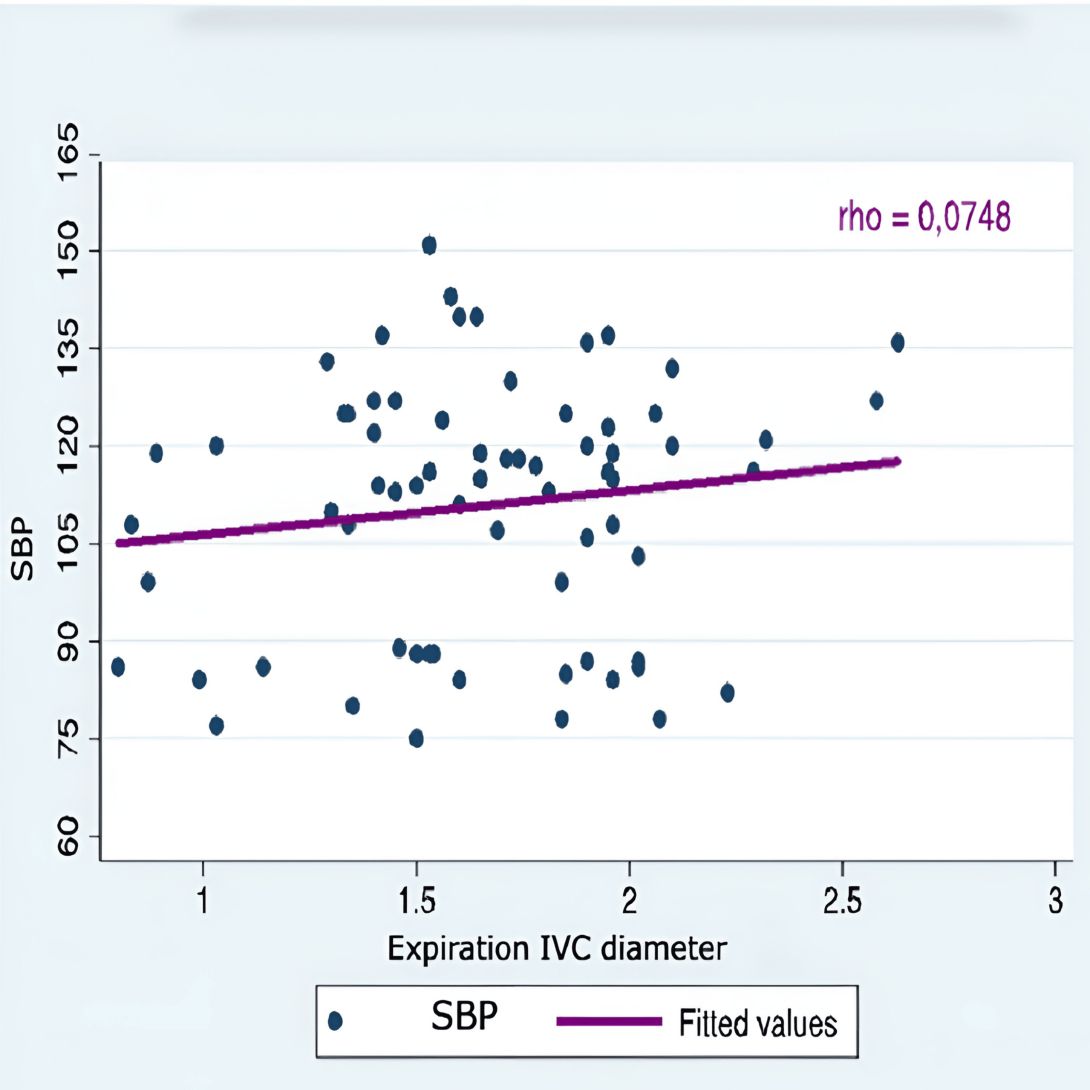
Correlation SBP and expiration IVC diameter SBP: systolic blood pressure; IVC: inferior vena cava; Y axis: SBP (mmHg); X axis: expiration IVC diameter (cm)

**Figure 3:**
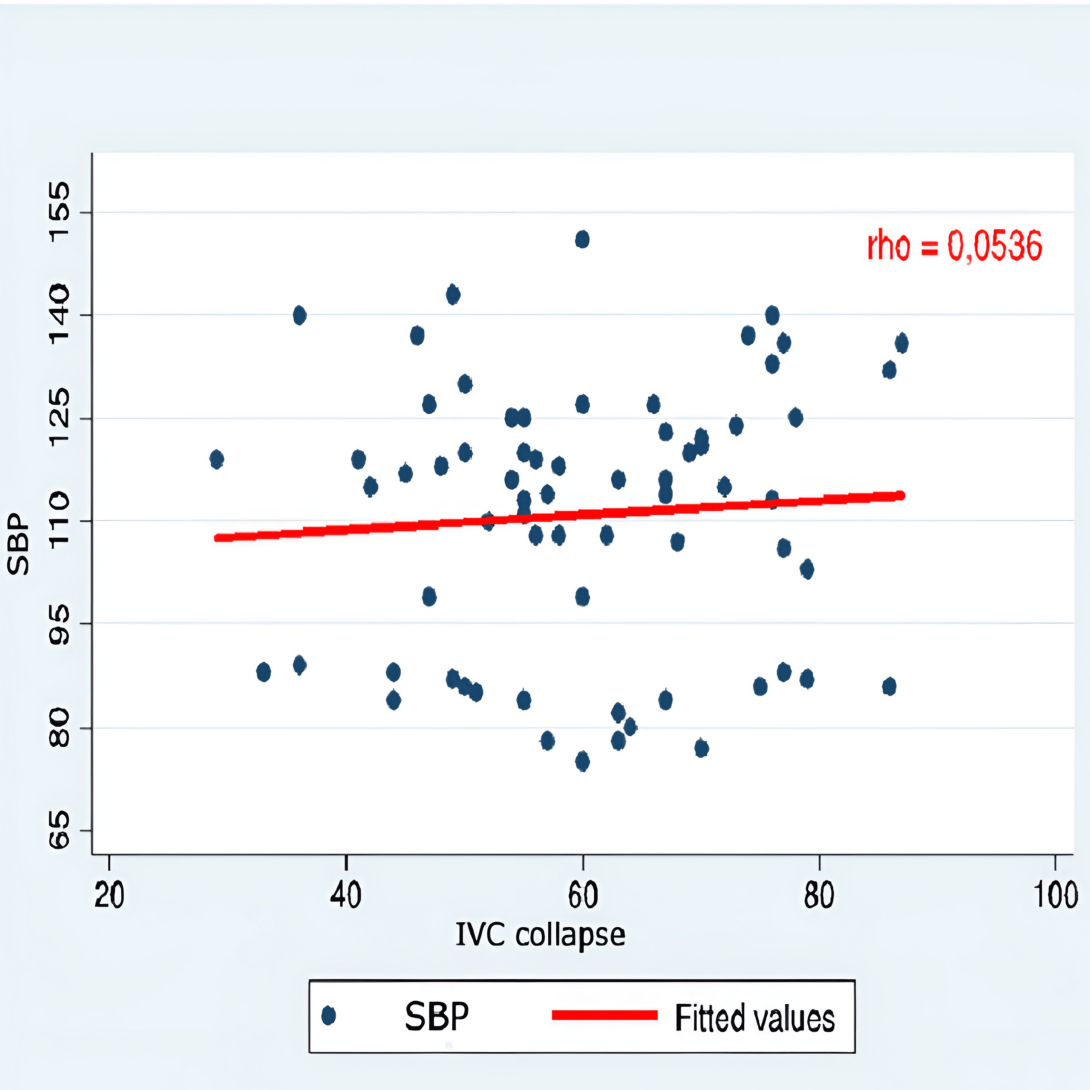
Correlation SBP and IVC collapse SBP: systolic blood pressure; IVC: inferior vena cava; Y axis: SBP (mmHg); X axis: IVC collapse (percentage %)

**Figure 4:**
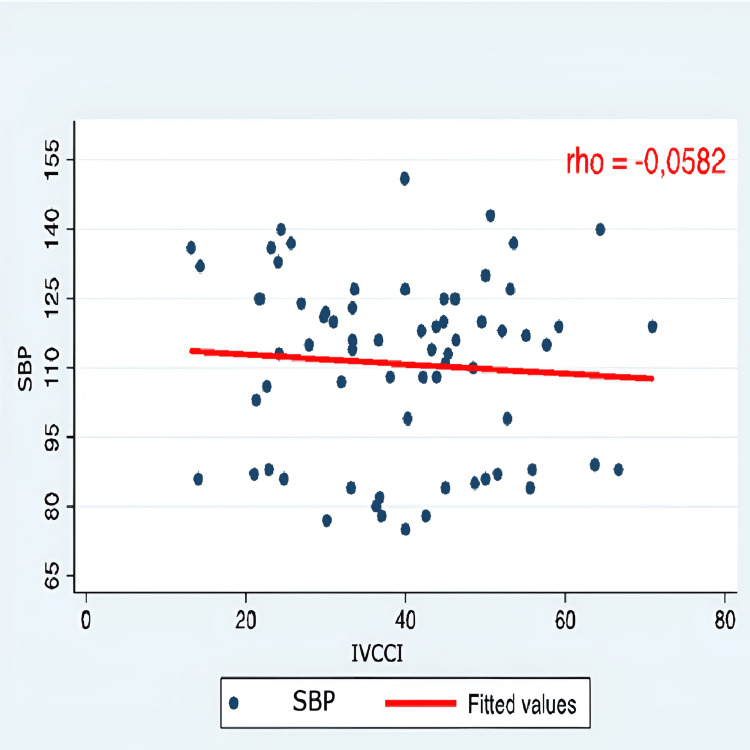
Correlation SBP and IVCCI SBP: systolic blood pressure; IVCCI: inferior vena cava collapsibility index; Y axis: SBP (mmHg); X axis: IVCCI (percentage %)

**Figure 5:**
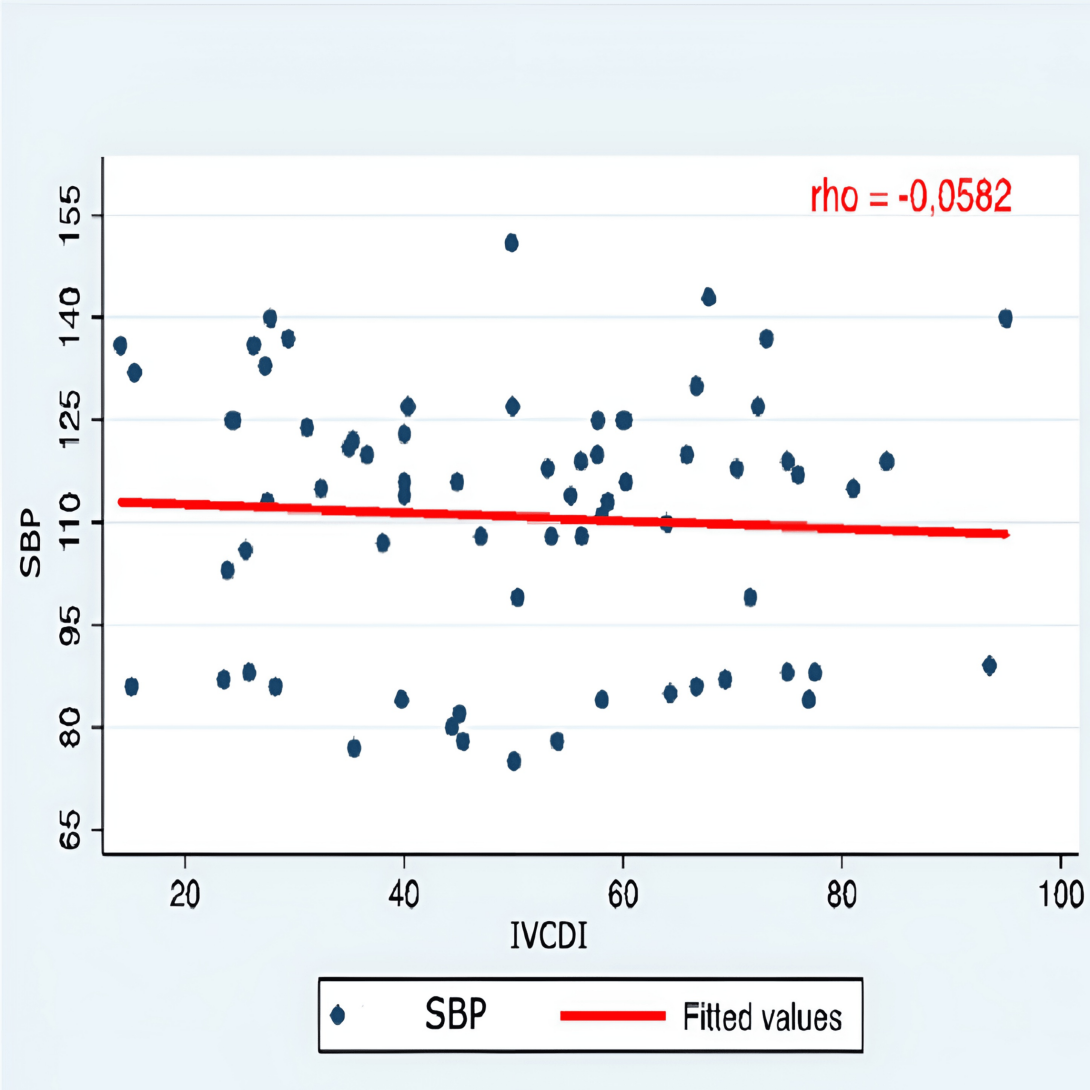
Correlation SBP and IVCDI SBP: systolic blood pressure; IVCDI: inferior vena cava distensibility index; Y axis: SBP (mmHg); X axis: IVCDI (percentage %)

Correlation graphs were created, considering only SBP, as there was no statistical significance in either SBP or MAP values. The evaluation of the correlation between arterial hypotension and monitoring IVC-PD using M-mode ultrasonography did not show a significant correlation with the variation in SBP. Therefore, it was concluded that IVC-PD cannot predict the occurrence of arterial hypotension (Figures 1-5).

## Discussion

The overall incidence of arterial hypotension in this study was 27.14% (95% CI: 16.42-37.82) (Table 3), showing a trend similar to the upper range of previously documented descriptive studies, which report an incidence between 15% and 33% in nonobstetric populations [1-3]. According to this study, hemodynamic changes were most frequently observed between minutes three and nine, which aligns with previously documented evidence [12, 13].

Several risk factors for hypotension have been described. These include a sensory block level above T5 (OR, 3.8), age 40 years or older (OR, 2.5), baseline SBP lower than 120 mmHg (OR, 2.4), and spinal puncture above L3-L4 (OR, 1.8) [1,2]. Other factors that influence the cardiovascular impact related to hypotension include the pharmacological characteristics of the medication used, such as latency, which increases the risk for drugs with short latencies [2]. Likewise, the patient’s physical condition and comorbidities are factors that determine the extent of BP changes. Clinical conditions such as hypovolemia, cardiovascular diseases, and a history of arterial hypertension amplify the hemodynamic impact. Anemia and obesity also contribute to this effect compared to healthy patients [14, 15].

In this study, the factors associated with the occurrence of arterial hypotension were consistent with previous investigations. As reported in the study by Crithley et al. [13], a history of arterial hypertension was associated with a higher risk of BP reduction during spinal anesthesia. Another statistically significant factor for the development of this event was a sensory block level above T4, aligning with findings previously documented in the literature [1].

This study offers several strengths, including its prospective design, adherence to standardized echocardiography guidelines for IVC measurements, and validation of known risk factors for hypotension, such as high sensory block levels (≥T4) and pre-existing arterial hypertension. These robust methodological aspects enhance the reliability of our findings while highlighting the need for further research to clarify the role of IVC assessment in managing spinal anesthesia. However, some limitations should be acknowledged. The modest sample size of 70 patients may affect generalizability, and a larger cohort could strengthen statistical power. As a single-center study, our results might reflect local practice patterns rather than broader clinical realities. Additionally, while having a single operator perform all IVC measurements ensured consistency, it precluded evaluation of inter-observer variability. Future multicenter studies employing standardized protocols and blinded multiple operators would help validate these findings and improve their clinical applicability.

We did not find a correlation that would establish IVC-PD as a reliable predictor of arterial hypotension in patients undergoing SAB. This result is consistent with the findings of Özdemir et al. [16], who also found no statistical significance between IVC diameter and the prediction of arterial hypotension. Similarly, in their research, Maciulene et al. [17] did not find utility in predicting intraoperative hypotension and bradycardia through the measurement of IVCIC in patients undergoing elective knee joint replacement surgery under SAB. However, in a cohort of nonobstetric patients receiving SAB in England, it was documented that the use of the IVCCI through ultrasonography is an effective method for preventing arterial hypotension and objectively guiding the requirement for crystalloid co-loading in this population [18].

However, the results reported by Aslan et al. [19] in their study of patients over 65 years old undergoing urological, general, orthopedic, plastic, and reconstructive surgeries differ. In their investigation, it was concluded that measuring IVC-PD via US before SAB in older adults is effective in predicting arterial hypotension.

The divergence in results among the various studies is attributed to several factors. Internal and external variables may affect the capacity of this venous vessel. Additionally, individual elasticity characteristics, influenced by factors such as a history of chronic diseases and age, may also impact measurements [20]. Alertness with spontaneous ventilation has been regarded as a relevant factor. This is due to differences in intrathoracic pressures compared to critically ill patients under mechanical ventilation, where breathing is a controlled and constant process [21]. This allows for the potential use of US-IVC-PD as a significant indicator for determining the patient’s volume status.

## Conclusions

This study confirms that arterial hypotension after SAB is influenced by well-established risk factors, including high sensory block levels, older age, lower baseline SBP, and a history of arterial hypertension. However, IVC measurements did not reliably predict hypotension, contrasting with some previous studies. These discrepancies may stem from variations in patient physiology, spontaneous ventilation effects, and differences in study populations. Further research is needed to clarify the role of IVC assessment, particularly in non-mechanically ventilated patients. Limitations such as sample size, single-operator ultrasound assessments, and single-center design underscore the need for larger, multicenter studies.
